# Medical education: future-proof?

**DOI:** 10.1007/s40037-013-0040-9

**Published:** 2013-01-31

**Authors:** Jan C. C. Borleffs

**Affiliations:** University Medical Center Groningen and University of Groningen, Groningen, the Netherlands

External site visits as part of the process of accreditation are an important aspect of quality control of medical education programmes. In 2011/2012 all medical schools (University Medical Centres, UMCs) in the Netherlands had such a visit. According to the accreditation system of the Netherlands and Flanders, accreditation has to be renewed every 6 years. The preparation of the site visit starts with the writing of a ‘critical reflection’ describing three aspects of the programme: the intended learning outcomes, the teaching and learning environment and the assessment. Furthermore, the critical reflection should present facts and figures about numbers of staff involved, supportive personnel, study results, etc. Due to changes in the accreditation system, the process of quality control of the programme is no longer part of the critical reflection and site visit. In the new system of accreditation the universities can apply for accreditation of the quality control process of all the programmes they offer [1]. Once institutional accreditation has been acquired, the site visits of the individual programmes are limited to the three standards mentioned above. Accreditation is given by the Netherlands and Flanders Accreditation Organization (NVAO) on the basis of the site visit report of an independent and certified visiting organization. As the Deans of the eight UMCs felt that this series of site visits should have additional value for all UMCs, the visiting organization was asked to evaluate all programmes in a separate ‘state of the art’ report. In autumn 2012, the state of the art report was presented [2]. The report contains examples of best practices, a benchmarking of the Dutch programmes with the curricula of medical schools abroad and the committee’s ideas about medical education in the next decades. The authors will elaborate in more detail on the content of the report in one of the forthcoming issues of PME.

In general, according to the opinion of the visiting committee, the quality of the medical programmes in the Netherlands is good and the graduates of these programmes meet the international standards of quality for medical doctors. However, although Dutch medical schools are very prominent in the area of research in medical education [3] and are at the frontline of innovation, the visiting committee suggests that a new step forwards should be made. The committee poses the challenging—but also rhetoric—question whether medical education is future-proof. This question fits very well with the growing opinion that both medical (undergraduate and graduate) education and postgraduate medical specialist training should get prepared for the next change. They refer to the recent publications of the Carnegie Foundation for the Advancement of Teaching [4] and The Lancet [5] and make a plea for a more system-based approach to medical education: after the concept of a science-based medical education, followed by the concept of problem-based education, it is now time for a system-based approach. Definitely, educational concepts based on previous approaches with proven effectiveness should be preserved, but the content of the programme needs to change in concurrence with the changes in society and the requirements for good health care in the next decades. Patient safety, multidisciplinary collaboration, health care for elderly people, leadership, efficiency and cost reduction of health care are characteristics of a more system-based approach to medical education. In the Dutch situation with the CanMEDS model as the national guideline for the content of the programmes of medical education, these themes can be implemented within this model [6]. In my opinion, the CanMEDS model offers a good framework for making medical education more future-proof.

Interestingly, in the process of implementing the CanMEDS model in postgraduate medical specialist training, the same themes have been identified for the education of the ‘intrinsic’ CanMEDS roles (communicator, organizer, collaborator, scholar, health advocate and professional). In the Netherlands, current experience with the CanMEDS roles in postgraduate training shows that the role of the medical expert is very prominent in the training programmes. The intrinsic roles, however, do not yet receive sufficient attention in the programmes. Last year, in order to help residents and medical specialist programme directors with the implementation of the intrinsic roles, the CanBetter project was started [7]. Just as we teach our students and residents the importance of livelong learning, the content of medical curricula and postgraduate specialist training programmes should continuously reflect the changes in society. In order to keep our programmes future-proof the plea of the visiting committee’s state of the art report inspires us to a livelong improvement of medical education.

The year of 2012 has meant a great change for *Perspectives on Medical Education*, the journal of the Netherlands Association of Medical Education. It was the first volume of the journal to be published in English. When we started the preparations for the change from a Dutch into an English journal, the great challenge was to increase the interest of authors and readers. So far, taking the number of submissions into account, the new format is a success. The number of submitted manuscripts has quadrupled and around 25 % of the first authors originate from countries outside the Netherlands and Flanders. At the same time the percentage of rejected manuscripts has increased to 30 %. In summary, after 1 year of experience with PME, it appears that the new format of the journal really appeals to a greater audience and facilitates the improvement in the quality of the journal. The recent inclusion of PME into the PubMed database will definitely contribute to the growth and strength of the journal.

With respect to the Editorial Board some changes will occur in 2013. According to the journal’s history, at present the members of the Editorial Board originate from the Netherlands and Flanders. However, it is our aim to change the Editorial Board gradually into a more international Board. Furthermore, as you can see in the advertisement in this issue of PME, there will be a vacancy for the position of Editor as—after more than two terms of being the Editor—I will be leaving this position in the course of 2013.
